# Supplementary material for: COVID-19 Vaccine Uptake Inequality Among Children: A Multidimensional Demographic Analysis
Source: Healthcare (Basel). 2025 Apr 29;13(9):1019. doi: 10.3390/healthcare13091019 (PMC12071228; doi:10.3390/healthcare13091019)

Supplementary Table S1. Frequencies and percentages of COVID-19 vaccination among the 16–17 years old in Jefferson County, Kentucky, by dose number, sex, and race on 5/31/2022

	Dose One			Dose Two		
	Both Sexes	Female	Male	Both Sexes	Female	Male
Frequencies						
All Races and Ethnicities	13,120	6,858	6,262	11,464	6,001	5,463
American Indian or Alaska Native	25	11	14	20	11	9
Asian	487	241	246	425	212	213
Black	2,465	1,290	1,175	2,086	1,094	992
Multiracial	1,503	783	720	1,364	709	655
Native Hawaiian or Other Pacific	14	6	8	9	3	6
Some Other Races	643	347	296	538	297	241
White	7,983	4,180	3,803	7,022	3,675	3,347
Hispanic or Latino	1,226	651	575	997	531	466
Non-Hispanic	11,894	6,207	5,687	10,467	5,470	4,997
Horizontal Percentages						
All Races and Ethnicities	100%	52.27%	47.73%	100%	52.35%	47.65%
American Indian or Alaska Native	100%	44.00%	56.00%	100%	55.00%	45.00%
Asian	100%	49.49%	50.51%	100%	49.88%	50.12%
Black	100%	52.33%	47.67%	100%	52.44%	47.56%
Multiracial	100%	52.10%	47.90%	100%	51.98%	48.02%
Native Hawaiian or Other Pacific	100%	42.86%	57.14%	100%	33.33%	66.67%
Some Other Races	100%	53.97%	46.03%	100%	55.20%	44.80%
White	100%	52.36%	47.64%	100%	52.34%	47.66%
Hispanic or Latino	100%	53.10%	46.90%	100%	53.26%	46.74%
Non-Hispanic	100%	52.19%	47.81%	100%	52.26%	47.74%
Vertical Percentages						
All Races and Ethnicities	100%	100%	100%	100%	100%	100%
American Indian or Alaska Native	0.19%	0.16%	0.22%	0.17%	0.18%	0.16%
Asian	3.71%	3.51%	3.93%	3.71%	3.53%	3.90%
Black	18.79%	18.81%	18.76%	18.20%	18.23%	18.16%
Multiracial	11.46%	11.42%	11.50%	11.90%	11.81%	11.99%
Native Hawaiian or Other Pacific	0.11%	0.09%	0.13%	0.08%	0.05%	0.11%
Some Other Races	4.90%	5.06%	4.73%	4.69%	4.95%	4.41%
White	60.85%	60.95%	60.73%	61.25%	61.24%	61.27%
Hispanic or Latino	9.34%	9.49%	9.18%	8.70%	8.85%	8.53%
Non-Hispanic	90.66%	90.51%	90.82%	91.30%	91.15%	91.47%

Supplementary Table S2. Frequencies and percentages of COVID-19 vaccination among the 12–15 years old in Jefferson County, Kentucky, by dose number, sex, and race on 5/31/2022

	Dose One			Dose Two		
	Both Sexes	Female	Male	Both Sexes	Female	Male
Frequencies						
All Races and Ethnicities	25,880	12,868	13,012	22,715	11,362	11,353
American Indian or Alaska Native	43	26	17	33	19	14
Asian	967	478	489	876	433	443
Black	5,057	2,525	2,532	4,270	2,140	2,130
Multiracial	3,394	1,684	1,710	3,067	1,527	1,540
Native Hawaiian or Other Pacific	28	12	16	24	10	14
Some Other Races	1,469	730	739	1,135	575	560
White	14,922	7,413	7,509	13,310	6,658	6,652
Hispanic or Latino	2,495	1,237	1,258	2,050	1,023	1,027
Non-Hispanic	23,385	11,631	11,754	20,665	10,339	10,326
Horizontal Percentages						
All Races and Ethnicities	100%	49.72%	50.28%	100%	50.02%	49.98%
American Indian or Alaska Native	100%	60.47%	39.53%	100%	57.58%	42.42%
Asian	100%	49.43%	50.57%	100%	49.43%	50.57%
Black	100%	49.93%	50.07%	100%	50.12%	49.88%
Multiracial	100%	49.62%	50.38%	100%	49.79%	50.21%
Native Hawaiian or Other Pacific	100%	42.86%	57.14%	100%	41.67%	58.33%
Some Other Races	100%	49.69%	50.31%	100%	50.66%	49.34%
White	100%	49.68%	50.32%	100%	50.02%	49.98%
Hispanic or Latino	100%	49.58%	50.42%	100%	49.90%	50.10%
Non-Hispanic	100%	49.74%	50.26%	100%	50.03%	49.97%
Vertical Percentages						
All Races and Ethnicities	100%	100%	100%	100%	100%	100%
American Indian or Alaska Native	0.17%	0.20%	0.13%	0.15%	0.17%	0.12%
Asian	3.74%	3.71%	3.76%	3.86%	3.81%	3.90%
Black	19.54%	19.62%	19.46%	18.80%	18.83%	18.76%
Multiracial	13.11%	13.09%	13.14%	13.50%	13.44%	13.56%
Native Hawaiian or Other Pacific	0.11%	0.09%	0.12%	0.11%	0.09%	0.12%
Some Other Races	5.68%	5.67%	5.68%	5.00%	5.06%	4.93%
White	57.66%	57.61%	57.71%	58.60%	58.60%	58.59%
Hispanic or Latino	9.64%	9.61%	9.67%	9.02%	9.00%	9.05%
Non-Hispanic	90.36%	90.39%	90.33%	90.98%	91.00%	90.95%

Supplementary Table S3. Frequencies and percentages of COVID-19 vaccination among the 5–11 years old in Jefferson County, Kentucky, by dose number, sex, and race on 5/31/2022

	Dose One			Dose Two		
	Both Sexes	Female	Male	Both Sexes	Female	Male
Frequencies						
All Races and Ethnicities	25,213	12,381	12,832	21,008	10,319	10,689
American Indian or Alaska Native	46	23	23	39	19	20
Asian	1,351	659	692	1,143	545	598
Black	4,153	2,059	2,094	3,180	1,592	1,588
Multiracial	3,377	1,652	1,725	2,897	1,420	1,477
Native Hawaiian or Other Pacific	40	24	16	27	16	11
Some Other Races	1,793	904	889	1,303	670	633
White	14,453	7,060	7,393	12,419	6,057	6,362
Hispanic or Latino	2,127	1,058	1,069	1,474	726	748
Non-Hispanic	23,086	11,323	11,763	19,534	9,593	9,941
Horizontal Percentages						
All Races and Ethnicities	100%	49.11%	50.89%	100%	49.12%	50.88%
American Indian or Alaska Native	100%	50.00%	50.00%	100%	48.72%	51.28%
Asian	100%	48.78%	51.22%	100%	47.68%	52.32%
Black	100%	49.58%	50.42%	100%	50.06%	49.94%
Multiracial	100%	48.92%	51.08%	100%	49.02%	50.98%
Native Hawaiian or Other Pacific	100%	60.00%	40.00%	100%	59.26%	40.74%
Some Other Races	100%	50.42%	49.58%	100%	51.42%	48.58%
White	100%	48.85%	51.15%	100%	48.77%	51.23%
Hispanic or Latino	100%	49.74%	50.26%	100%	49.25%	50.75%
Non-Hispanic	100%	49.05%	50.95%	100%	49.11%	50.89%
Vertical Percentages						
All Races and Ethnicities	100%	100%	100%	100%	100%	100%
American Indian or Alaska Native	0.18%	0.19%	0.18%	0.19%	0.18%	0.19%
Asian	5.36%	5.32%	5.39%	5.44%	5.28%	5.59%
Black	16.47%	16.63%	16.32%	15.14%	15.43%	14.86%
Multiracial	13.39%	13.34%	13.44%	13.79%	13.76%	13.82%
Native Hawaiian or Other Pacific	0.16%	0.19%	0.12%	0.13%	0.16%	0.10%
Some Other Races	7.11%	7.30%	6.93%	6.20%	6.49%	5.92%
White	57.32%	57.02%	57.61%	59.12%	58.70%	59.52%
Hispanic or Latino	8.44%	8.55%	8.33%	7.02%	7.04%	7.00%
Non-Hispanic	91.56%	91.45%	91.67%	92.98%	92.96%	93.00%

Supplementary Table S4. P-values from proportion tests assessing vaccination inequality across age groups for each dose on 5/31/2022

	05–11 vs 12–15	12–15 vs 16–17	05–11 vs 16–17
Dose1	<0.001	<0.001	<0.001
Dose2	<0.001	<0.001	<0.001

Supplementary Table S5. P-values from proportion tests assessing vaccination inequality between female and male, White and other racial groups, and Hispanic and non-Hispanic groups within age group by dose on 5/31/2022

	Female vs. Male	White vs. Black	White vs. Asian	White vs. Multiracial	White vs. Some Other Races	Hispanic vs. Non-Hispanic
05–11						
Dose1	0.277	<0.001	0.027	<0.001	<0.001	<0.001
Dose2	0.320	<0.001	0.191	<0.001	0.098	<0.001
12–15						
Dose1	<0.001	<0.001	<0.001	0.538	0.376	<0.001
Dose2	<0.001	<0.001	<0.001	0.607	<0.001	<0.001
16–17						
Dose1	<0.001	<0.001	<0.001	<0.001	0.205	<0.001
Dose2	<0.001	<0.001	0.001	<0.001	0.004	<0.001

Supplementary Figure S1. Trends in one and two-dose COVID-19 vaccine uptake by age and race in Jefferson County, Kentucky (numbers in thousands)

Dose One

Dose Two

(a) 16–17

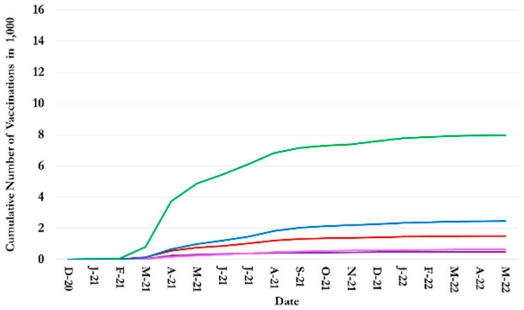

(b) 16–17

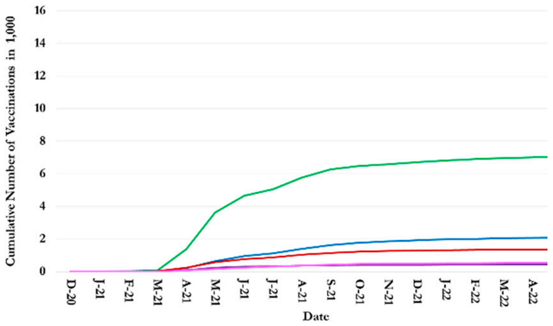

(c) 12–15

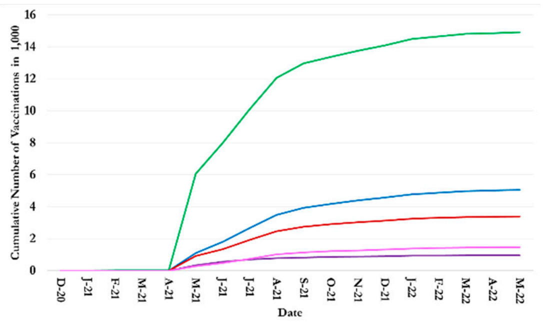

(d) 12–15

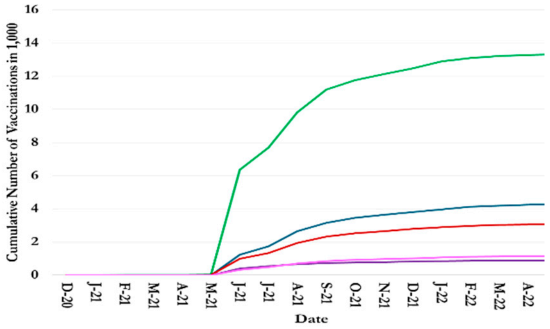

(e) 5–11

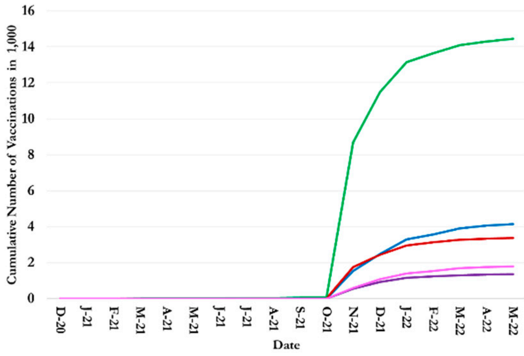

(f) 5–11

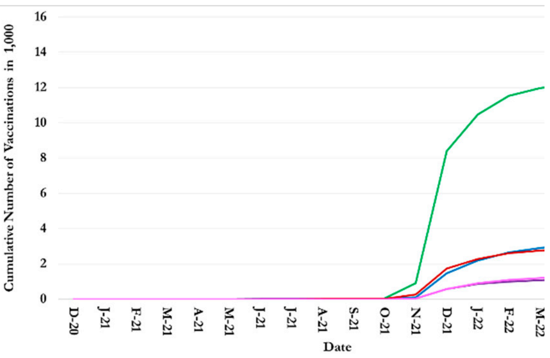

Black White Asian Multiracial Some other races

Supplementary Figure S2. Trends in one and two-dose COVID-19 vaccine uptake by age and ethnicity in Jefferson County, Kentucky (numbers in thousands)

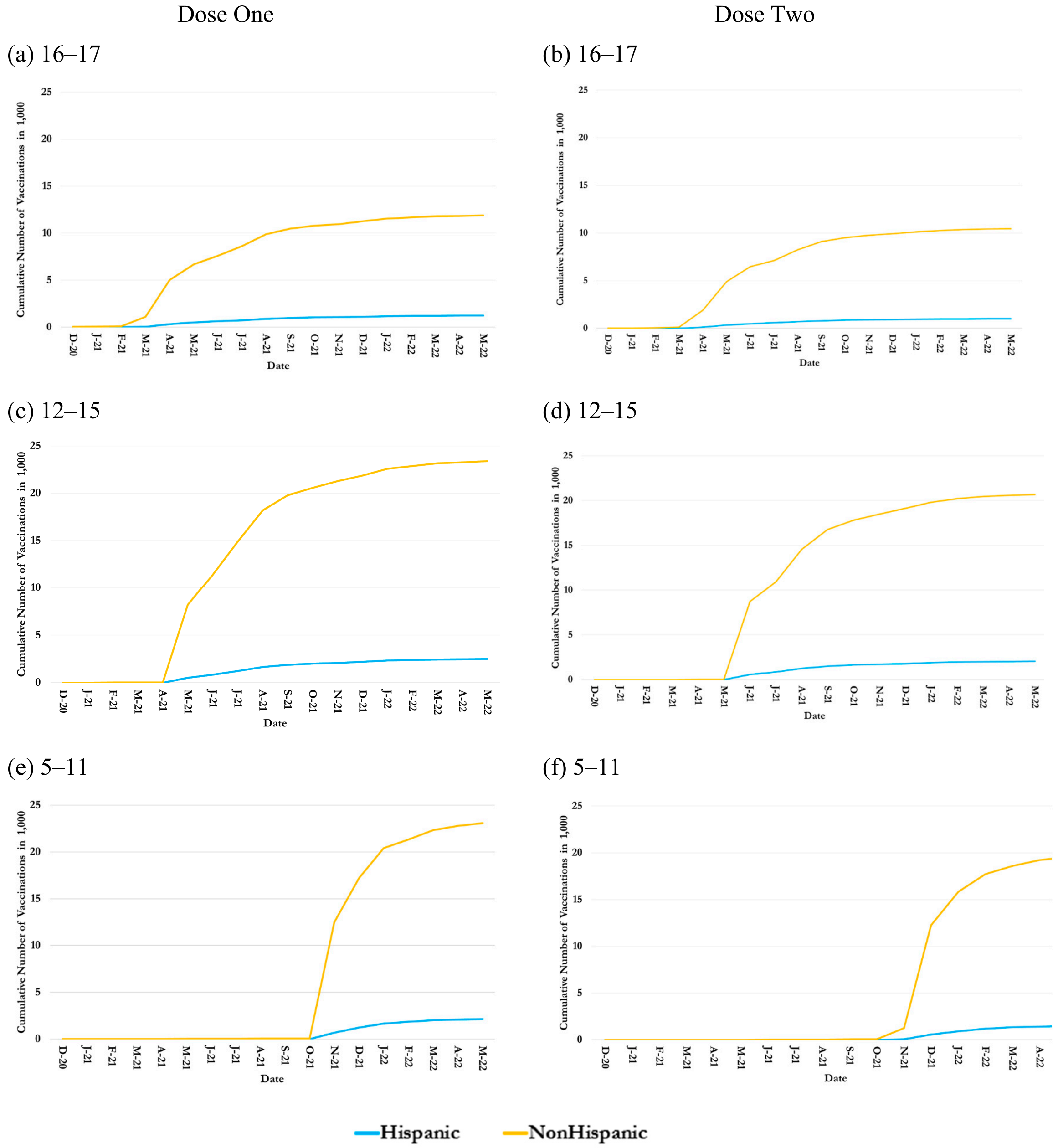

Supplement: Supplementary file 1 [file healthcare-13-01019-s001.zip › healthcare-3553706-supplementary.pdf]
